# Enhancing Yellow Pea Protein Extraction and Purification Through Ultrafiltration

**DOI:** 10.3390/membranes15110326

**Published:** 2025-10-28

**Authors:** Muhammad Nurdarwis Bin Anuar, Jian Zuo

**Affiliations:** Food, Chemical and Biotechnology, Singapore Institute of Technology, 1 Punggol Coast Road, Singapore 828608, Singapore; muhammadnurdarwis.binanuar@singaporetech.edu.sg

**Keywords:** protein extraction, ultrafiltration, protein yield, protein purity, water efficiency

## Abstract

The growing demand for sustainable, high-quality plant-based proteins has increased the need for efficient extraction and purification methods for yellow pea protein (*Pisum sativum* L.). Conventional techniques, such as isoelectric precipitation (IEP) and wet fractionation, often result in moderate protein recovery (50–70%), reduced functionality, and high water consumption. This study evaluates ultrafiltration (UF) as a mild, membrane-based alternative for yellow pea protein extraction. Under optimized conditions, UF achieved protein recovery above 85% while maintaining high solubility (>90%) and emulsification capacity. Additionally, incorporating water recycling into the UF process reduced total water use by up to 60%. These results demonstrate that UF offers a more efficient and environmentally sustainable approach for producing functional yellow pea protein compared with traditional methods.

## 1. Introduction

Yellow pea protein (*Pisum sativum* L., YPP) has attracted attention as a sustainable, versatile plant protein thanks to its good nutritional profile and wide functional applications [1,2]. Not only does this legume improve soil fertility through nitrogen fixation [3] but it also requires less water and agrochemical input than many other protein crops. Nutritionally, YPP is rich in lysine and arginine, and—when blended with cereal proteins—it delivers a balanced essential amino acid profile [3]. Functionally, its excellent solubility, emulsification, gelation, and foaming capacities underpin its use in dairy alternatives, meat extenders, high-protein beverages, and bakery products [4,5]. Together, these attributes explain why YPP is worth scaling up, spurring development of extraction and purification methods that maximize yield, maintain functionality, and reduce environmental impact.

The extraction of YPP encompasses a range of processing techniques, each developed to enhance protein yield, preserve or improve functional attributes, and address growing concerns surrounding environmental sustainability [6,7]. Among these, conventional methods such as IEP and wet fractionation have been extensively applied at both laboratory and industrial scales. IEP is a widely used technique that involves adjusting the pH of an aqueous protein extract to the isoelectric point (typically around pH 4.5 for pea proteins), at which the net charge of the proteins becomes neutral, resulting in aggregation and precipitation [1,7]. The precipitated protein is then separated from the liquid phase by centrifugation, followed by neutralization and drying steps. This method is straightforward, scalable, and capable of producing protein isolates with purities ranging from 75% to 85% [7,8]. However, protein recovery is often limited, with yields typically reported between 50% and 70%, depending on extraction conditions and pea variety [4,9].

Despite its widespread use, IEP frequently results in proteins with compromised functional properties due to the extreme pH shifts and subsequent neutralization steps involved in the process. These conditions can cause partial denaturation, aggregation, and loss of solubility—key attributes required for applications in emulsions, beverages, and meat analogs [2,10]. Furthermore, IEP generates large volumes of acidified wastewater that require additional treatment before disposal or reuse, contributing to environmental burden and increased treatment costs. In contrast, wet fractionation—though milder—still requires substantial amounts of water and often yields protein fractions of lower purity, necessitating further downstream processing. Together, these limitations have prompted the development and evaluation of alternative technologies such as membrane-based separations, which offer the potential for improved protein recovery, better preservation of native functionality, and enhanced water efficiency.

In recent years, UF has been applied to YPP separation, delivering consistently high recovery rates. Mondor, Tuyishime, and Drolet [11] evaluated different UF modules and reported YPP recoveries between 81.8% and 83.8%. Earlier, Mondor et al. [12] demonstrated that defatted pea acidic extraction followed by UF achieved even higher recoveries of 85.7–88%, with isolates maintaining excellent functional properties. Beyond YPP, UF has also been employed for other legume proteins: Boye et al. [1] found that chickpea and lentil protein isolates processed via UF retained recoveries above 80% and preserved solubility and emulsification capacities. These findings confirm that UF not only concentrates plant proteins efficiently but also preserves their native functionality across multiple sources.

Furthermore, UF facilitates water recycling during the extraction process, addressing environmental concerns associated with large-scale water consumption [13,14]. By integrating UF with sustainable practices—such as permeate recirculation—water usage can be reduced by up to 60% without compromising protein yield or quality. Consequently, UF-based fractionation is driving the evolution of the YPP industry toward scalable, resource-efficient production methods that meet both performance and sustainability targets.

Building upon these developments, our modified UF runs has shown promise in addressing water usage and product recovery concerns, as it operates under relatively mild conditions and does not rely on harsh chemical treatments. By employing a semi-permeable membrane with a carefully selected pore size, UF effectively separates high-molecular-weight protein fractions from low-molecular-weight compounds [5,15]. As a result, the extracted protein remains largely intact, retaining its native structure and functional properties such as solubility, emulsification, and gelation. In contrast to IEP, which necessitates significant pH adjustments and generates chemical-laden effluents, our experiments permits the reclaiming and recycling of process water, thereby reducing the overall water footprint [13,14]. This water recovery capability not only aligns with sustainable production goals but also translates to cost savings associated with both water procurement and waste disposal. Consequently, our scaled-up UF research presents a viable strategy for enhancing protein yield and purity while simultaneously minimizing environmental impact and preserving the intrinsic qualities of YPP.

In this study, a process based on UF was developed and evaluated for the extraction and purification of YPP. Following initial solubilization, a mild heat treatment was applied to facilitate protein extraction, after which UF was used to concentrate and purify the protein fractions. For comparison, IEP was also conducted as a baseline purification method. This involves precipitating the extracted solution at 1 M hydrochloric acid (HCl), allowing for the separation of non-soluble protein components. Additionally, permeate water generated during UF was recovered and reused in the extraction and washing steps. The results demonstrated that UF achieved higher protein recovery and purity than IEP, and these metrics remained essentially unchanged when water recycling was implemented. Thus, this study highlights the potential of UF as a sustainable and effective method for YPP production, offering valuable insights into enhancing protein yield, quality, and resource utilization.

## 2. Materials and Methods

### 2.1. Materials

Yellow pea flour (YPF) was obtained from Agrocorp International Pte. Ltd. (Singapore). UF was carried out using the Sartoflow^®^ Advanced system equipped with Sartocon^®^ Tangential Flow Filtration (TFF) ultrafiltration cassettes (Sartorius Singapore Pte. Ltd., Singapore). The specifications of the membrane cassettes used can be found in Table 1. All chemicals, sodium hydroxide and hydrochloric acid, used in the study were purchased from Sigma-Aldrich Pte. Ltd. (Singapore).

### 2.2. Protein Extraction Procedure

**Figure 1:**
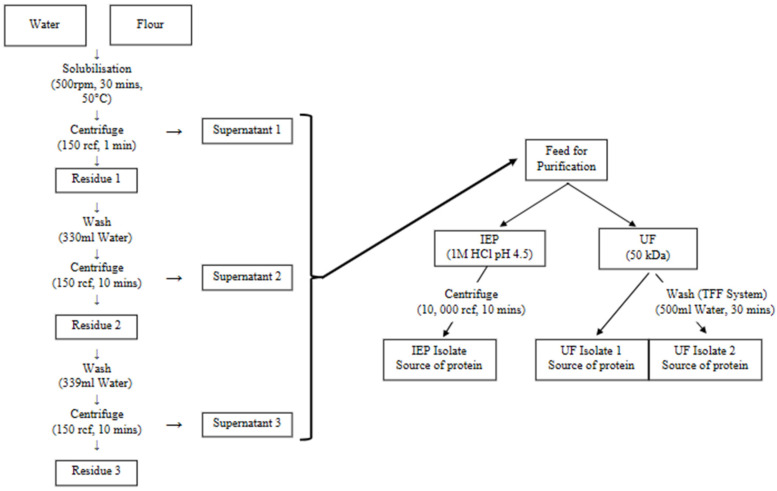
Process flow from extraction and purification to protein collection.

Heat Fractionation was performed for protein extraction, which included one round of suspension followed by two rounds of washing. YPF was dispersed in water at a 1:4 *w*/*w* ratio, i.e., 100 g YPF in 400 mL of water. The extraction was performed at 50 °C in a water bath for 30 min. After extraction, the dispersion underwent the first centrifugation step at 150 rcf for 1 min using a floor centrifuge (Thermo Scientific™ Sorvall™ LYNX 4000, Thermo Fisher Scientific, Waltham, MA, USA). During this step the insoluble starch granules were separated into the pellet. The supernatant was collected for further purification steps. The pellet was then washed with 330 mL of deionized (DI) water and hand-stirred for 1 min to aid dispersion. Then, the dispersion was submitted to a second round of centrifugation at 150 rcf for 10 min. The resulting supernatant was combined with the initial supernatant, while the pellet underwent another round of washing under the same conditions. The final combined supernatant was used for the purification stage. Figure 1 shows the schematic of the process described.

### 2.3. Purification: IEP and UF

**Figure 2:**
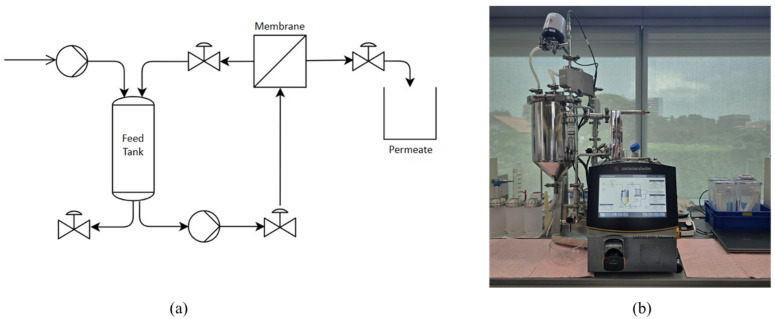
(**a**) process flow diagram and (**b**) picture of the TFF System Sartoflow^®^ Advanced.

The supernatant obtained from extraction was firstly centrifuged at 10,000× *g* for 10 min. The pellet from this centrifuge was freeze-dried and stored as protein fraction, whereas the supernatant was used for further purification using two methods: IEP at pH 4.5 and UF. For IEP, the pH of the supernatant solution was adjusted to 4.5 by gradually adding 1 M hydrochloric acid. Then, the solution was centrifuged at 10,000 rcf for 10 min, separating the precipitate protein as a pellet. For UF, the process was carried out using a Tangential Flow Filtration System (Sartoflow^®^ Advanced TFF System, Göttingen, Germany) with a 50 kDa membrane (surface area of 0.1 m^2^, Sartocon^®^ Polyethersulfone TFF Ultrafiltration Cassettes, Göttingen, Germany). Figure 2a shows the process flow diagram of the TFF system and Figure 2b shows the actual image of the system. UF step was conducted at a volume concentration ratio (VCR) of 10, a transmembrane pressure (TMP) of 1 bar, temperature of 25 °C ± 2 °C and feed flowrate of 10 L/h. The system was operated under manufacturer-recommended conditions, as detailed crossflow channel dimensions were not available for velocity estimation. The retentate was then collected as the protein isolate. Following UF, the TFF system was washed with 500 mL of DI water for 30 min. The wash water, containing residual protein, was then collected as an additional protein source. The pellet from IEP, protein isolate from UF and wash water were oven-dried and resultant protein was stored at 4 °C for further analysis.

All protein extraction and purification experiments were performed in triplicate (n = 3). Protein recovery and purity were measured for each replicate, and the average values are reported.

### 2.4. Water for Extraction

To reduce water consumption during the YPP extraction process, permeate water collected after each UF cycle was evaluated for its potential reuse in subsequent extraction steps. Three experimental models were established: (1) full DI water was used for extraction, (2) a 1:1 mixture of DI water and permeate water, and (3) full permeate water. Each model was subjected to identical centrifugation and purification parameters (using the 50 kDa membrane) to ensure consistency in extraction efficiency across the different water sources. This approach facilitated the assessment of the impact of varying water compositions on the yield and quality of YPP, while also allowing for the quantification of total water consumption during the extraction process.

**Figure 3:**
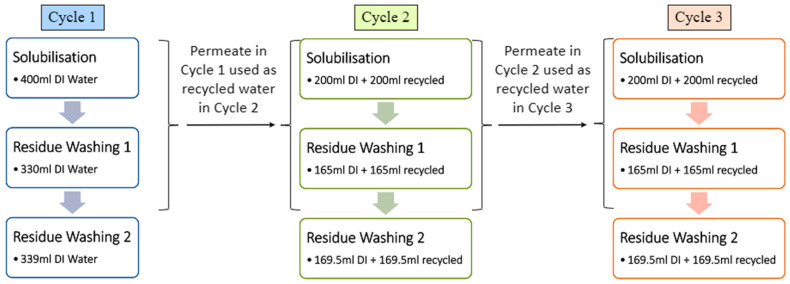
Water usage with half recycled for second and third consecutive runs.

As illustrated in Figure 3, each extraction cycle was carried out independently using a fresh batch of YPF. The first extraction cycle used only DI water, and the resulting permeate collected from UF was reused in the subsequent extraction as a 1:1 mixture of DI water and permeate. This approach was repeated for later cycles, with arrows in Figure 3 indicating the origin and reuse of permeate water from preceding runs. Over time, the volume of permeate generated becomes sufficient to support extraction using only permeate water, thereby reducing overall DI water consumption while maintaining comparable extraction performance.

### 2.5. Determination of Protein Content

Total nitrogen content in the dried samples was determined using a Kjeldahl digestion system (DUMATHERM N Pro, Königswinter, Germany). To estimate total protein content, a nitrogen-to-protein conversion factor of 6.25 was applied, as recommended for legume proteins [16]. For each analysis, 0.2 g of dried sample was used, and all measurements were performed in triplicate.

The protein content of each sample was calculated using the following equation:

Protein Content (g) = Nitrogen Content (g) × 6.25

The protein purity was then calculated as the proportion of protein in the total dry sample:

$$\mathrm{Protein}\;\mathrm{Purity}\;(\%)=\frac{Protein\;Content\;(g)}{Dry\;Sample\;Weight\;(g)}\times 100$$

To determine protein recovery, the protein content obtained from each dried fraction was compared to the total protein in the YPF used for extraction:

$$Protein\;Recovery\;(\%)=\frac{Protein\;Content\;in\;Dried\;Fractions\;(g)}{Total\;Protein\;in\;extracted\;flour\;(g)}\times 100$$

### 2.6. Protein Characterization

Sodium Dodecyl Sulfate Polyacrylamide Gel Electrophoresis (SDS-PAGE) was performed to evaluate the protein profiles of Yellow Pea Isolate (YPI) obtained through UF and IEP. Samples were prepared using 2× Laemmli buffer, denatured at 95 °C for 5 min, and loaded at 10 µL per lane. The gels were stained with Coomassie Brilliant Blue G-250 and imaged using the Bio-Rad ChemiDoc MP system (Bio-Rad Laboratories, Hercules, CA, USA) equipped with a white light conversion screen under UV illumination.

### 2.7. Membrane Performance Evaluation

The performance of the UF membrane was assessed through several key parameters. The flux was calculated by using the sample weight collected at 10 min interval throughout the total concentration period divided by the area of membrane and converted to L/m^2^-h.

For comparative evaluation, membranes with molecular weight cut-offs (MWCO) of 10, 30, and 50 kDa were tested under identical operating conditions to examine the effect of membrane pore size on protein transmission and recovery.

$$Flux=\frac{m}{A\times t}$$

where m is the mass (kg) of permeate sample collected over time t (h) and *A* is the effective membrane area in m^2^, which is 0.1 m^2^ for the membrane cassette used.

Membrane permeability was calculated according to the following equation.

$$P=\frac{Flux}{∆P}$$

where Δ*P* is the transmembrane pressure.

The volume concentration ratio (*VCR*) was calculated with the equation

$$VCR=\frac{V_{i}}{V_{f}}$$

where *V_f_* is the final volume of the concentrate and *V_i_* is the initial volume of the feed solution, providing insights into the concentration effects on membrane performance.

### 2.8. Permeate Characterization

The protein content of the UF permeate was assessed using the Bradford Assay, a colorimetric method that utilizes Coomassie Brilliant Blue dye to quantify protein concentration. In this assay, 100 µL of the permeate sample was mixed with 900 µL of Bradford reagent, followed by incubation at room temperature for 15 min to allow for the formation of a stable dye-protein complex. The absorbance of the resulting solution was measured at 595 nm using a spectrophotometer (GENESYS™ UV-Vis Spectrophotometer, Thermo Fisher Scientific Inc., Waltham, MA, USA). To quantify the protein content, a standard curve was constructed using known concentrations of bovine serum albumin (BSA). The concentration of protein was calculated based on the linear relationship between absorbance and concentration established by the standard curve.

## 3. Results and Discussions

### 3.1. Protein Extraction Evaluation

Möller et al. [17] demonstrated that additional pellet washing steps enhance protein enrichment in mild wet fractionation by removing interstitial water containing solubilized proteins and other components. Based on these findings, multiple washing steps were incorporated into the heat fractionation process to improve protein recovery and purity.

Following initial solubilization, two pellet washing steps were performed using low centrifugal force to minimize protein loss in the supernatant which is collected for purification. As previously hypothesized by Möller et al. [17], additional pellet washing steps were expected to improve protein recovery by removing interstitial soluble proteins. After two sequential centrifugation–wash cycles, a high protein recovery of 92.96% was achieved in the supernatant. This indicates that additional wash steps confer only marginal gains in recovery while substantially increasing water consumption. Thus, limiting the protocol to two washes provides a practical balance between yield and water conservation.

### 3.2. Protein Purification

Following protein extraction through the heat fractionation process, downstream purification was carried out using either IEP or UF. Table 2 shows the comparison of the two methods in terms of protein recovery and purity. UF, using 50 kDa membrane, achieved a protein recovery rate of approximately 83%, with protein purity reaching 78%, as measured using the DUMATHERM system. This improvement is attributed to UF’s ability to retain high-molecular-weight protein fractions while removing low-molecular-weight impurities through selective membrane separation. In contrast, IEP resulted in lower protein recovery of approximately 68% with comparable purity. It has been observed that, at pH 4.5, acid-soluble pea protein fractions remain in the supernatant rather than precipitating, thereby reducing overall recovery [12]. Consequently, IEP recovers only the acid-insoluble proteins, leaving the soluble fraction unrecovered and limiting total protein yield [1]. The results show the superior performance of UF in achieving higher recovery and comparable protein purity.

### 3.3. UF Membrane Performance

Protein recovery from UF was also influenced by membrane selection and operation conditions. The performance parameters of the UF system during protein purification were determined based on conditions widely reported in the literature, with membrane selection and pre-treatment steps chosen to ensure consistent and reliable operation. To optimize the performance of the UF system during protein purification, membrane selection and pre-treatment steps were carefully considered. Three membranes with MWCO of 10 kDa, 30 kDa and 50 kDa were evaluated in terms of permeability and flux.

**Figure 4:**
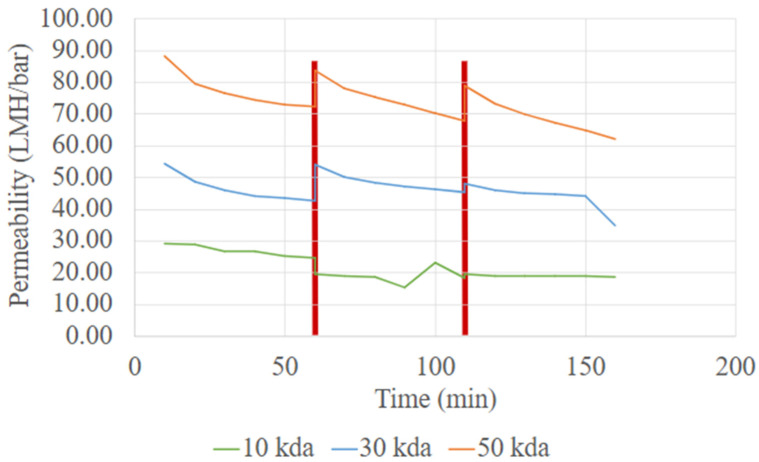
Membrane permeability data for varying membrane MWCO. (Red lines indicate the time points at which the membranes were backwashed before resuming operation).

Figure 4 shows the variation in permeability over time for 10 kDa, 30 kDa and 50 kDa membranes during the concentration of the extraction solution. A simple backwash of membrane was conducted after 60 min of operation. The permeability trend follows the order of MWCO, with the 50 kDa membrane exhibiting the highest permeability, and the 10 kDa membrane the lowest. This trend is attributed to difference in membrane pore size, where large pore size facilitates faster permeate flow [18]. Higher permeability is desirable in the UF process, as it reduces processing time, energy consumption, and required membrane area, ultimately leading to lower operational and membrane costs.

The 50 kDa membrane was selected for the UF process development because it achieves a balance between high throughput and acceptable protein retention for the targeted YPP [19]. However, as shown in Figure 4, the 50 kDa membrane also exhibits a greater decline in permeability over time compared to the 30 kDa and 10 kDa membranes, indicating a higher susceptibility to membrane fouling [20]. The larger pore can allow the entry and penetration of macromolecular aggregates and suspended solids, leading to partial pore blocking and reduced membrane performance. Despite this, with the simple backwash, permeability can be largely recovered. Thus, the 50 kDa membrane remains a strong candidate for YPP purification due to its operational efficiency and compatibility with process-scale throughput requirements. With appropriate pre-treatment steps (such as centrifugation to clarify feed solutions) and routine membrane cleaning, the long-term performance of the 50 kDa membrane can be maintained.

Thus, to further protect the 50 kDa membrane and enhance UF membrane performance, an additional centrifugation step was introduced following protein extraction. Building on the water-only fractionation protocol of [17], which employs multiple washing and centrifugation steps to separate yellow peas into starch-rich, soluble protein-rich, and non-soluble protein-rich fractions, a modified two-step centrifugation was implemented in the study. Following initial extraction, the suspension was centrifuged at 10,000× *g* for 20 min to sediment the starch-rich fraction. The supernatant was then subjected to a second 10,000× *g*, 20 min spin to partition it into a soluble protein-rich phase and a non-soluble protein-rich pellet. This approach produced a clarified feed for UF, substantially reducing suspended solids, minimizing membrane fouling, and enhancing permeate flux and membrane longevity. Additionally, removing insoluble contaminants prior to UF improved the purity of the final protein concentrate by eliminating non-protein particulates. Overall, membrane performance and protein recovery were improved through both appropriate membrane selection and the strategic inclusion of centrifugation as a pre-filtration step.

### 3.4. SDS-PAGE Analysis of Protein Isolates

**Figure 5:**
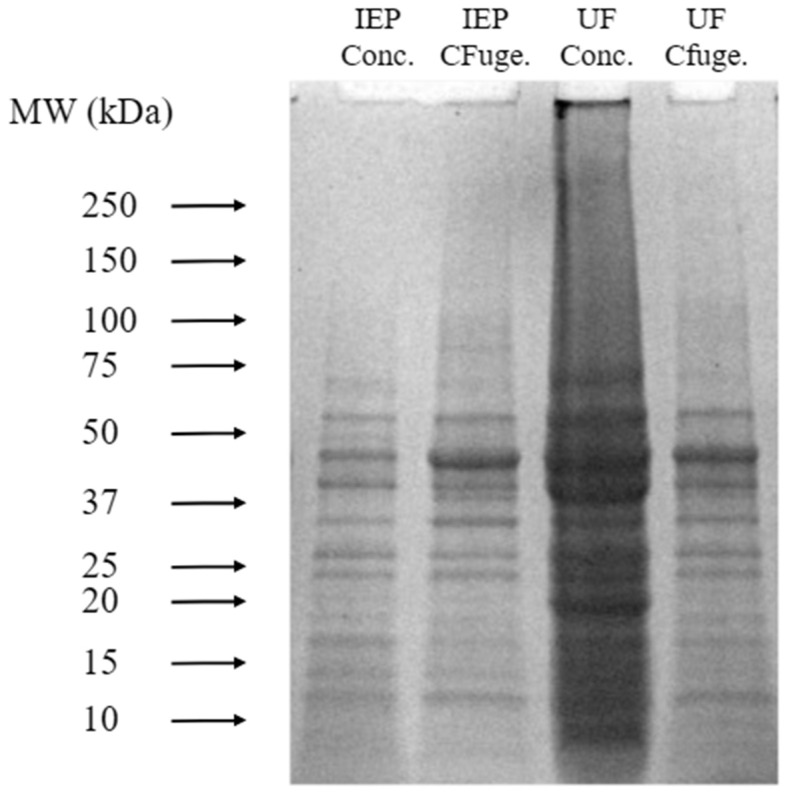
SDS-Page patterns of yellow pea protein isolates obtained from different purification steps.

Figure 5 shows SDS-PAGE patterns of yellow pea protein isolates obtained from different purification steps: (1) the final precipitate from IEP, referred to as IEP concentrate (IEP conc.); (2) the supernatant collected after centrifugation and before IEP, labeled as IEP centrifuge (IEP CFuge.); (3) the final concentrate from UF, referred to as UF concentrate (UF conc.); and (4) the supernatant collected after centrifugation and prior to UF, labeled as UF centrifuge (UF CFuge.). The gel patterns provide insight into the molecular weight distribution and purity of the protein fractions resulting from each purification step.

The UF lanes exhibited higher band intensity with some degree of smearing, which may result from concentrated protein loading rather than aggregation or denaturation. This observation reflects the efficiency of UF in enriching soluble protein fractions. Although SDS-PAGE cannot directly infer the preservation of native tertiary structure, the distinct and continuous banding across a broad molecular weight range suggests that UF effectively retained soluble and diverse protein components. This aligns with findings by [21], who reported similar protein subunit profiles in legume isolates subjected to membrane-based separations.

In comparison, the IEP lanes appeared clearer but less intense, indicating lower soluble protein recovery. The reduced band intensity likely reflects partial loss of soluble proteins during acid precipitation, while the sharper bands correspond to a narrower range of acid-insoluble protein fractions. This agrees with previous studies showing that IEP can lead to selective precipitation of proteins with reduced solubility under acidic conditions, potentially limiting recovery of functional fractions.

Additionally, the supernatant samples collected after centrifugation—but before IEP or UF—displayed comparable SDS-PAGE profiles, confirming that the upstream extraction process consistently solubilized yellow pea proteins. Hence, the observed differences in the final concentrates can be attributed primarily to the subsequent purification method rather than the extraction step itself.

After processing, IEP concentrates exhibited brighter, more intense bands for specific fractions, likely due to enrichment of insoluble globulin subunits or enhanced Coomassie dye binding caused by partial unfolding under acidic conditions. In contrast, the UF concentrate, though more diffuse, represents a higher overall soluble protein content, consistent with the concentration and retention mechanisms of ultrafiltration.

Across all samples, reduced intensity above ~75 kDa was observed, which is typical of legume protein isolates dominated by vicilin and legumin subunits. High-molecular-weight fractions may have been partially lost during processing or precipitated under acidic conditions.

Overall, the SDS-PAGE results highlight that UF and IEP differ fundamentally in their separation mechanisms. While IEP yields denser, more selective fractions through precipitation, UF offers a milder, concentration-based process that retains a broader spectrum of soluble proteins. These characteristics make UF more suitable for applications requiring high solubility and functional integrity in plant-based formulations.

### 3.5. Water Usage

The UF process demonstrated a clear advantage in water recovery and reuse compared to IEP in the context of YPP extraction. Three water usage models were tested to evaluate both water savings and their effects on protein recovery: (1) full use of DI water, (2) a 1:1 mixture of DI and recycled permeate water, and (3) full use of recycled permeate water. For the extraction of 200 g of YPF, the base case (full DI water) required approximately 1.5 L of water for extraction and washing. By integrating permeate recycling, the total DI water requirement was reduced to 0.75 L (in the mixed model) and 0 L (in the full permeate model), with the latter relying entirely on previously recovered UF permeate. However, due to system losses (e.g., membrane hold-up and evaporation), fresh DI water was periodically needed to top up the recycled stream, especially in long runs. Therefore, full permeate model was tested primarily to demonstrate feasibility rather than as a sustainable standalone model.

**Figure 6:**
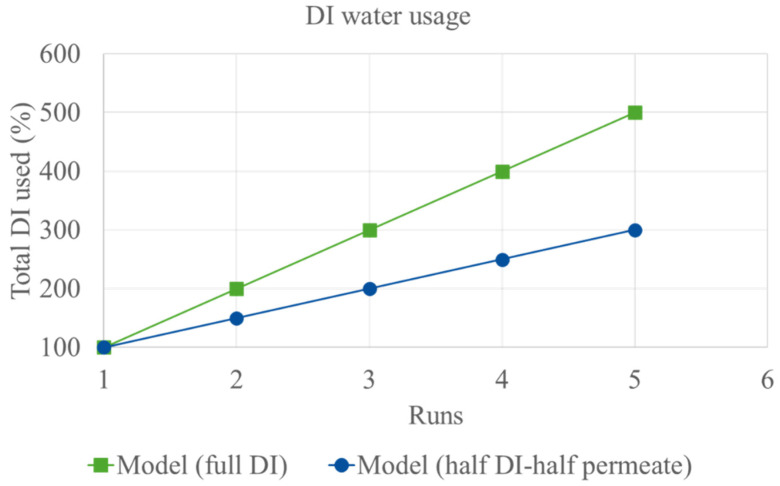
Theoretical projection of DI usage with full DI and half DI-half permeate water across the runs.

Importantly, protein recovery remained consistent across all water models, as shown in Table 3. Recovery using full DI water was approximately 83%, while the mixed model and full permeate model yielded 85% and 87%, respectively. Similarly, protein purity showed only minor variations, with full DI water at approximately 77%, the mixed model at 79%, and the full permeate model at 78%, suggesting that using recycled water had minimal impact on extraction efficiency. This supports the viability of recycled permeate as a sustainable water source without compromising protein recovery. Table 2 shows the comparison of water usage, protein recovery, and protein purity across different water recycling strategies in the UF process. Figure 6 illustrates the projected DI water usage under the full DI model and the 1:1 mixed model. With just 1 run of recycling, the 1:1 mixed model achieves 25% reduction in water usage. As the number of recycling cycles increases, the percentage of water saved continues to grow. Furthermore, the ratio between fresh DI water and recycled permeate can be adjusted in practical applications, allowing for flexible optimization of water usage based on process requirements and sustainability goals.

In contrast, IEP is not compatible with water recycling due to the introduction of hydrochloric acid (HCl) during the pH adjustment step. The resulting aqueous effluent contains high levels of dissolved salts and residual chemicals, which render the water unsuitable for reuse without extensive treatment. This chemically contaminated waste stream not only prevents water recovery but also increases disposal requirements, operational costs, and environmental burden. Therefore, while UF supports water efficiency through effective permeate recycling, IEP produces a non-recyclable by-product stream, limiting its sustainability in large-scale protein processing.

## 4. Conclusions

UF demonstrated clear advantages over IEP in the purification of YPP, achieving higher protein recovery and purity while improving water reuse efficiency. Unlike IEP, which produces chemically altered wastewater unsuitable for recycling, UF enabled the recovery of permeate water for subsequent extraction cycles, substantially reducing fresh water demand. The mild operating conditions of UF also helped preserve the solubility and functional integrity of YPP, as confirmed by SDS-PAGE analysis.

Additionally, UF showed potential for process intensification through sequential membrane operation and scale-up, which can further enhance purity and recovery efficiency. While membrane fouling, cleaning frequency, and scalability remain practical challenges, the overall findings confirm UF as a sustainable and effective approach for high-quality plant protein purification. Future studies should focus on optimizing membrane performance and long-term operation to support industrial implementation.

## Figures and Tables

**Table 1:** Characteristics of Sartocon^®^ Ultrafiltration Cassettes.

Casette Format	Slice Cassettes
Flow Channel Geometry	E-Screen (High Viscosity)
Membrane Material	Polyethersulfone (PES)
Molecular weight cut-off (MWCO)	50
Filtration mode	Tangential Flow
Surface area (m^2^)	0.1

**Table 2:** Composition of YPP in different purification conditions.

Purification Conditions	pH	Protein Recovery (%)	Protein Purity (%)
Wet Fractionation (i.e., only extraction step with no further purification)	7	92.96	68.15
IEP	4.5	67.89	78.90
UF Full DI (50 kDa membrane)	7	82.78	77.81

**Table 3:** Water usage, protein recovery, and protein purity of yellow pea protein extracted using ultrafiltration under different water recycling strategies.

Water Usage Model	DI Water Used (L)	Recycled Permeate Used (L)	Protein Recovery (%)	Protein Purity (%)
**Full DI water**	1.5	0	83	77
**Mixed (1:1 DI:permeate)**	0.75	0.75	85	79
**Full permeate**	0	1.5	87	78

## Data Availability

Data is contained within the article.
